# Applications of Copolymers Consisting of 2,6-di(9H-carbazol-9-yl)pyridine and 3,6-di(2-thienyl)carbazole Units as Electrodes in Electrochromic Devices

**DOI:** 10.3390/ma12081251

**Published:** 2019-04-16

**Authors:** Chung-Wen Kuo, Jui-Cheng Chang, Yu-Ting Huang, Jeng-Kuei Chang, Li-Ting Lee, Tzi-Yi Wu

**Affiliations:** 1 Department of Chemical and Materials Engineering, National Kaohsiung University of Science and Technology, Kaohsiung 80778, Taiwan; welly@nkust.edu.tw (C.-W.K.); a3473179@gmail.com (Y.-T.H.); 2 Bachelor Program in Interdisciplinary Studies, National Yunlin University of Science and Technology, Yunlin 64002, Taiwan; d700215@gmail.com; 3 Department of Materials Science and Engineering, National Chiao Tung University, Hsinchu 30010, Taiwan; jkchang@nctu.edu.tw; 4 Department of Materials Science and Engineering, Feng Chia University, Taichung 40724, Taiwan; ltlee@fcu.edu.tw; 5 Department of Chemical Engineering and Materials Engineering, National Yunlin University of Science and Technology, Yunlin 64002, Taiwan

**Keywords:** copolymer, electrochromic material, spectroelectrochemistry, coloration efficiency, electrochromic device, redox stability

## Abstract

A series of carbazole-based polymers (PdCz, P(dCz2-*co*-dTC1), P(dCz2-*co*-dTC2), P(dCz1-*co*-dTC2), and PdTC) were deposited on indium tin oxide (ITO) conductive electrodes using electrochemical polymerization. The as-prepared P(dCz2-*co*-dTC2) displayed a high Δ*T* (57.0%) and multichromic behaviors ranging from yellowish green, greenish gray, gray to purplish gray in different redox states. Five organic electrochromic devices (ECDs) were built using dCz- and dTC-containing homopolymers and copolymers as anodic materials, and poly(3,4-(2,2-dimethylpropylenedioxy)thiophene) (PProdot-Me_2_) as the cathodic material. The P(dCz2-*co*-dTC2)/PProdot-Me_2_ ECD presented remarkable electrochromic behaviors from the bleached to colored states. Moreover, P(dCz2-*co*-dTC2)/PProdot-Me_2_ ECD displayed a high optical contrast (Δ*T*, 45.8%), short switching time (ca. 0.3 s), high coloration efficiency (528.8 cm^2^ C^−1^) at 580 nm, and high redox cycling stability.

## 1. Introduction

Over the past numerous years, several inorganic and organic electrochromic materials have been extensively studied for use in the rear-view mirrors of vehicles, displays, helmet visors, and windows of buildings [1]. In these electrochromic materials, reversible redox reactions lead to an important change in transmitted (or reflected) light. The promising inorganic electrochromic materials are transition metal oxides (e.g., WO_3_, Ta_2_O_5_, TiO_2_, Nb_2_O_5_, and MoO_3_), whereas the potential organic electrochromic materials are π-conjugated polymers, viologen derivatives, metallophthalocyanines, and metallopolymers [2]. Among these organic electrochromic materials, recent research has concentrated interest on the applications of conjugated polymers as electrochromic (EC) electrodes due to their fast-electrochromic switching time [3], satisfactory coloration efficiency [4], and wide color availability through the chemical structures modification [5]. The organic EC materials are particularly polyaniline [6], polytriphenylamine [7], polyindole [8], polycarbazole [9], polypyrrole [10], polythiophene [11], polyselenophene [12], poly(3,4-ethylenedioxythiophene) (PEDOT) [13], and polyanthracene [14]. Polytriphenylamine, polycarbazole and their derivatives have been broadly used for numerous optical and electrochemical devices due to their good hole-transporting/mobile abilities and high thermal stability [15,16]. The redox peaks and electrochromic behaviors of polytriphenylamine and polycarbazole depend on the types and number of substituents, which contain electron-donating and electron-withdrawing units. Ak et al. reported on the electrochromic behaviors of a carbazole based star shaped polymer (PTPC). The PTPC displayed absorption peaks at 308, 460, and 780 nm, which are in agreement with the π-π* transition peak, polaron band, and bipolaron band of the PTPC, respectively. The PTPC film was green in its oxidized state and transparent in its reduced state [17]. Zhang et al. reported the preparation and electrochromic characterizations of a triphenylamine-, carbazole-, and EDOT-containing electrochromic copolymer film (P(CDPN-*co*-EDOT)) [18]. The P(CDPN-*co*-EDOT) film displayed four kinds of colors (claret red, green, cadetblue, and blue) from the neutral state to the oxidized state. The Δ*T* of P(CDPN-*co*-EDOT) was 36% and 43% in visible zone and near-IR region, respectively. Polythiophene and its derivatives are widely studied owing to their interesting characteristics such as narrow band gaps and desirable stability in air [19]. However, the onset oxidation potentials of polythiophene films are high [20]. Reynolds et al. synthesized the EDOT analogs such as 3,4-propylenedioxythiophene (ProDOT) and EDOT-Me and investigated the electrochromic properties of their corresponding PProDOT and PEDOT-Me polymer films, which displayed lower onset oxidation potentials than those of polythiophene films. The Δ*T* of these PEDOT derivatives were 44–63% at ca. 590 nm [21]. Since PProDOT film displays no significant absorption band in UV-Vis zone in oxidized state and the color is dark blue in reduced state, PProDOT is a potential cathodic material of electrochromic devices (ECDs). Moreover, vapor phase polymerization (VPP) and oxidative chemical vapor deposition (oCVD) are also widely used in synthesis of conjugated polymers [22,23].

In the present study, two homopolymers (PdCz and PdTC) and three copolymers (P(dCz2-*co*-dTC1), P(dCz2-*co*-dTC2), P(dCz1-*co*-dTC2)) with different dCz/dTC feed molar ratios were electrosynthesized to study their potential applications in ECDs. 2,6-di(9H-carbazol-9-yl)pyridine unit comprises two carbazole groups linked by a pyridine core, such donor-acceptor-donor (D-A-D) configuration is efficient for narrowing the band gap of polymers. Moreover, two carbazole groups in each repeat unit of polymer backbone facilitate the formation of radical cations and dication upon oxidation. In addition, the chemical structure of 3,6-di(2-thienyl)carbazole group displays that a carbazole unit inserts between two thiophene units, PdTC is easier to oxidize due to the donor capability of the carbazole. Poly(3,6-carbazole) has been shown to have a short conjugation length. The incorporation of thiophenes at 3,6-positions of carbazole unit would extend this conjugation length, leading to low oxidation potentials. Furthermore, five ECDs consisted of PdCz, P(dCz2-*co*-dTC1), P(dCz2-*co*-dTC2), P(dCz1-*co*-dTC2), or PdTC as the anodic material, and PProdot-Me_2_ as the cathodic material were fabricated and their spectroelectrochemical characteristics, transmittance-time profiles, and long-term electrochemical stability were studied in detail.

## 2. Experimental

### 2.1. Materials

2,6-di(9H-carbazol-9-yl)pyridine (dCz), 3,6-di(2-thienyl)carbazole (dTC), and Prodot-Me_2_ were prepared according to previously published methods [24,25]. Electrolytes of electrochromic devices were prepared using poly(methyl methacrylate) (PMMA):propylene carbonate (PC):LiClO_4_ in a weight ratio of 33:53:14 [26].

### 2.2. Electrochemical Preparation of PdCz, P(dCz2-co-dTC1), P(dCz2-co-dTC2), P(dCz1-co-dTC2), PdTC, and PProdot-Me_2_ Films

The electrosynthesis of PdCz, P(dCz2-*co*-dTC1), P(dCz2-*co*-dTC2), P(dCz1-*co*-dTC2), and PdTC films were carried out in an 0.2 M LiClO_4_/acetonitrile (ACN)/dichloromethane (DCM) (1:1, by volume) solution, and the feed ratio of species for anodic polymer films were listed in Table 1. The carbazole-based polymer films were electrodeposited potentiodynamically by sweeping voltages in a range of 0.0–1.4 V (vs. Ag/AgNO_3_) for 3 cycles. The Ag/AgNO_3_ electrode was calibrated using ferrocene. PProdot-Me_2_ film was electrodeposited potentiostatically at 1.0 V (vs. Ag/AgNO_3_). Polymeric thicknesses at the electrode surfaces obtained from an Alpha-Step profilometer (KLA Tencor D-120, Milpitas, CA, USA) were about 180–300 nm.

### 2.3. Fabrication of Electrochromic Devices

ECDs were constructed using PdCz, P(dCz2-*co*-dTC1), P(dCz2-*co*-dTC2), P(dCz1-*co*-dTC2), or PdTC film as the anodic layer and PProdot-Me_2_ film as the cathodic layer. The active areas of anode and cathode were 1.5 cm^2^. The ECDs were built by arranging the reduced and oxidized polymeric films to face each other, and they were isolated by a PMMA/PC/ACN/LiClO_4_ composite electrolyte.

### 2.4. Characterizations of Polymer Films and ECDs

The as-prepared polymer films and ECDs were characterized using a CHI627D electrochemical analyzer (CH Instruments, Austin, TX, USA) and an Agilent Cary 60 UV-Visible spectrophotometer (Varian Inc., Walnut Creek, CA, USA). The system of electrochemical experiments was implemented in a three-constituent cell. The working electrode was an ITO coated glass plate, the counter electrode was a platinum wire, and an Ag/AgNO_3_ electrode was used as the reference electrode. Spectroelectrochemical experiments were monitored using a spectrophotometer, the spectroelectrochemical characterizations of polymer films were performed in a UV quartz cuvette cell (Varian Inc., Walnut Creek, CA, USA), and the path length of cell was 1 cm.

## 3. Results and Discussion

### 3.1. Electrochemical Polymerization and FT-IR Characterization

**Figure 1:**
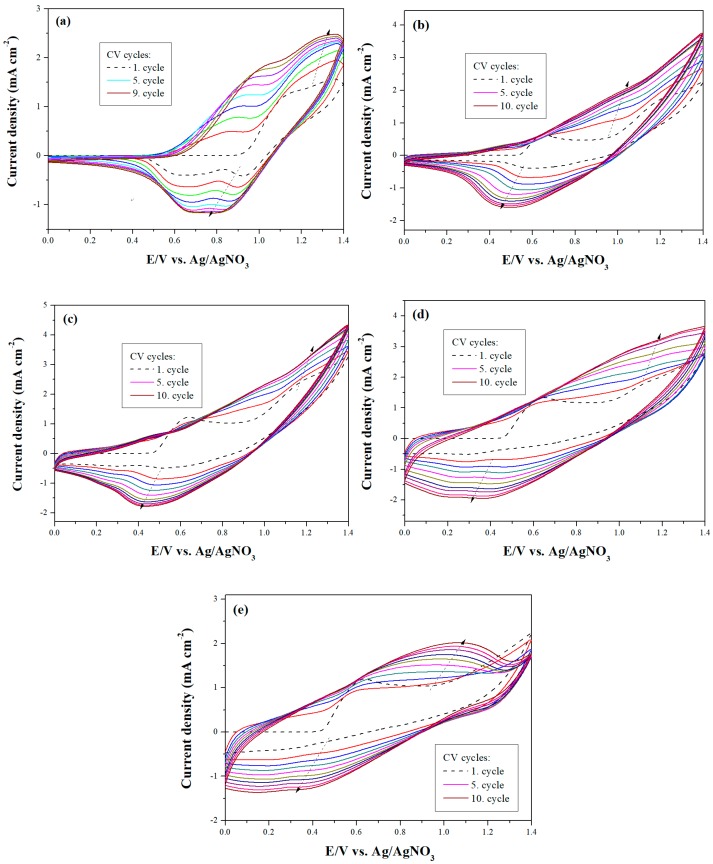
Electrochemical synthesis of (**a**) PdCz; (**b**) P(dCz2-*co*-dTC1); (**c**) P(dCz2-*co*-dTC2); (**d**) P(dCz1-*co*-dTC2) and (**e**) PdTC in ACN/DCM (1:1, by volume) solution at 100 mV s^−1^ on ITO working electrodes.

**Figure 2:**
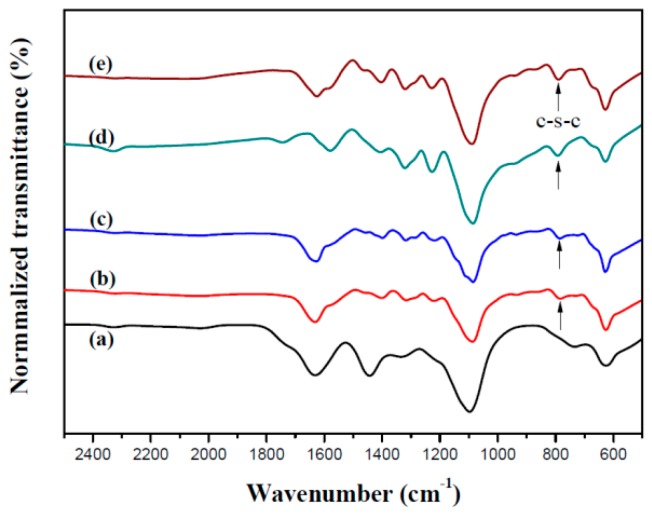
FT-IR spectra of (**a**) PdCz; (**b**) P(dCz2-*co*-dTC1); (**c**) P(dCz2-*co*-dTC2); (**d**) P(dCz1-*co*-dTC2) and (**e**) PdTC.

Figure 1 displays the cyclic voltammograms (CV) of 2 mM dCz, 2 mM dTC, and their mixtures (2 mM dCz + 1 mM dTC; 2 mM dCz + 2 mM dTC; 1 mM dCz + 2 mM dTC) in 0.2 M LiClO_4_/ACN/DCM solution in the potential range from 0.0 to 1.4 V. The scan rate was 100 mV s^−1^. When the number of CV cycles increased, the current density of redox peaks increased with an increasing scanning number, implying the growth of polymer films on ITO surfaces [27]. As displayed in Figure 1a, the 1st and 2nd oxidation peaks of PdCz at ca. 0.87 and 1.30 V (vs. Ag/AgNO_3_), respectively, can be ascribed to the presence of radical cation and dication in a dCz unit. The CV curves of PdTC show an oxidation and a reduction peaks at 1.05 and 0.37 V, respectively, as shown in Figure 1e. The electrochemical redox peaks of PdCz and PdTC were quasi-reversible. When the CV curves were swept in the mixture of dCz and dTC monomers, the oxidation peaks of P(dCz2-*co*-dTC1), P(dCz2-*co*-dTC2), and P(dCz1-*co*-dTC2) appeared at 1.25, 1.19 and 1.15 V, respectively, and the reduction peaks of P(dCz2-*co*-dTC1), P(dCz2-*co*-dTC2), and P(dCz1-*co*-dTC2) located at 0.50, 0.43 and 0.39 V, respectively, as shown in Figure 1b–d. The redox potentials and wave shapes of copolymers are different from those of homopolymers, and this is an evidence of the copolymerization of P(dCz2-*co*-dTC1), P(dCz2-*co*-dTC2), and P(dCz1-*co*-dTC2) films. The polymer films were further characterized using FT-IR, Figure 2 displayed the FT-IR spectra of electrochemically synthesized PdCz, P(dCz2-*co*-dTC1), P(dCz2-*co*-dTC2), P(dCz1-*co*-dTC2), and PdTC films. The characteristic peaks of PdCz are shown in Figure 2a. The characteristic band at 1095 cm^−1^ indicates the doping of PdCz film with the ClO_4_^−^. The characteristic peaks at around 1600 cm^−1^ represent the aromatic C=C stretching vibration. The band at 1220 cm^−1^ is related to C–C formation. The peak at around 1450 cm^−1^ can be ascribed to the C–N stretching of the carbazole unit [28]. 

**Figure 3:**
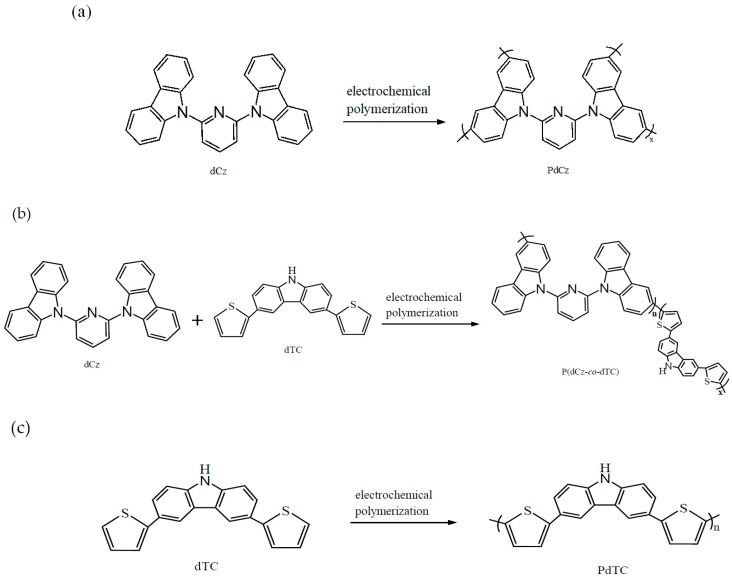
The electrochemical polymerization routes of (**a**) PdCz; (**b**) P(dCz-*co*-dTC) and (**c**) PdTC.

There was no conspicuous characteristic peak of PdCz at ca. 790 cm^−1^ as shown in Figure 2a. Figure 2b–d not only revealed the characteristic peaks of PdCz but also displayed the characteristic peak (-C-S-C- stretching) of PdTC, the formation of a new characteristic peak at 790 cm^−1^ could be attributed to the presence of dTC in P(dCz2-*co*-dTC1), P(dCz2-*co*-dTC2), and P(dCz1-*co*-dTC2) films, implying dCz- and dTC-containing copolymer films were successfully synthesized. The polymerization schemes of PdCz, P(dCz-*co*-dTC), and PdTC are shown in Figure 3.

### 3.2. Electrochemical Properties of Polymer Films

**Figure 4:**
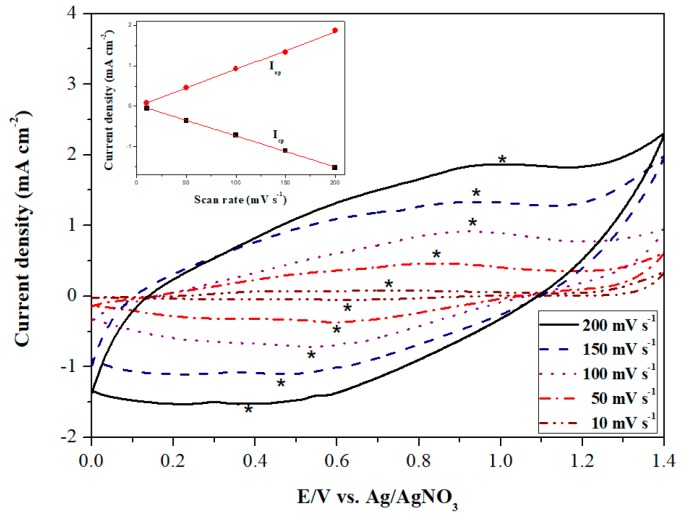
Cyclic voltammograms (CV) curves of the P(dCz2-*co*-dTC2) film at different scan rates between 10 and 200 mV s^−1^ in the LiClO_4_/ACN/DCM solution. Inset is scan rate dependence of the P(dCz2-*co*-dTC2) anodic and cathodic peak current densities.

The first rinse P(dCz2-*co*-dTC2) film was immersed into clean electrolyte solution, and then run in new CV curves. The as-prepared P(dCz2-*co*-dTC2) film was swept at 10, 50, 100, 150, and 200 mV s^−1^ in 0.2 M LiClO_4_/ACN/DCM using CV. As shown in Figure 4, the anodic and cathodic peaks of P(dCz2-*co*-dTC2) film showed a single quasi-reversible redox process and the anodic and cathodic peak current densities increased linearly with increasing scan rate (inset in Figure 4), implying the redox process of P(dCz2-*co*-dTC2) film was not a diffusion-controlled process [29].

### 3.3. Spectroelectrochemical Investigation of Polymer Films

**Figure 5:**
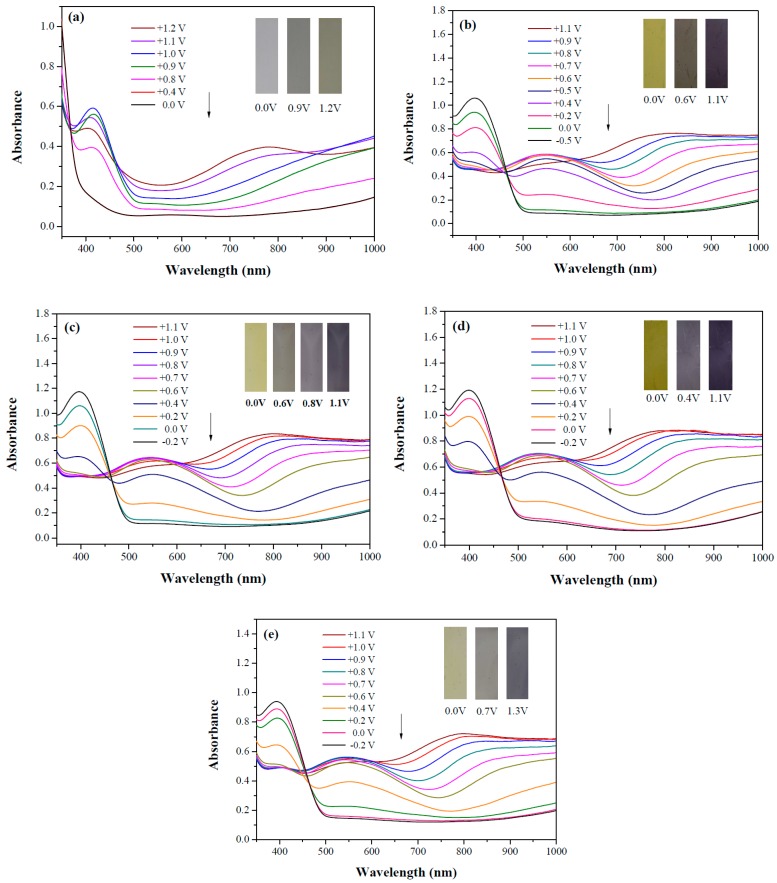
UV-Visible spectra of (**a**) PdCz; (**b**) P(dCz2-*co*-dTC1); (**c**) P(dCz2-*co*-dTC2); (**d**) P(dCz1-*co*-dTC2) and (**e**) PdTC electrodes on ITO in ACN/DCM (1:1, by volume) solution containing 0.2 M LiClO_4_.

Figure 5 shows UV-Visible spectra of PdCz, P(dCz2-*co*-dTC1), P(dCz2-*co*-dTC2), P(dCz1-*co*-dTC2), and PdTC electrodes in an ACN/DCM solution containing 0.2 M LiClO_4_. PdCz does not show distinct absorption peaks at 0.0 V but two new peaks appear at 430 and 785 nm gradually upon increasing of the applied potential, which can be attributed to the growth of charge carrier bands [30]. On the other hand, P(dCz2-*co*-dTC1), P(dCz2-*co*-dTC2), P(dCz1-*co*-dTC2), and PdTC electrodes display absorption peaks at 406, 410, 405, and 402 nm at 0.0 V, respectively, which can be assigned to the π-π* transition of P(dCz2-*co*-dTC1), P(dCz2-*co*-dTC2), P(dCz1-*co*-dTC2), and PdTC electrodes in their reduced state. When potentials in the oxidative direction were applied, the π-π* transition bands of P(dCz2-*co*-dTC1), P(dCz2-*co*-dTC2), P(dCz1-*co*-dTC2), and PdTC electrodes waned and new charge carrier bands (550 nm and 860 nm for P(dCz2-*co*-dTC1); 545 nm and 820 nm for P(dCz2-*co*-dTC2); 548 nm and 830 nm for P(dCz1-*co*-dTC2); 549 nm and 815 nm for PdTC) were seen at the longer wavelengths zone. The optical band gap (*E*_g_) values of PdCz, P(dCz2-*co*-dTC1), P(dCz2-*co*-dTC2), P(dCz1-*co*-dTC2), and PdTC electrodes were 3.01, 2.43, 2.48, 2.42, and 2.45 eV, respectively.

PdCz displayed multichromic behaviors with a light gray color in its neutral state (0.0 V), an iron grey color (0.9 V) and a dark gray color (1.2 V) during stepwise oxidation. The colors of PdTC electrode were light yellowish-gray, light gray, and iron grey at 0.0, 0.7, and 1.3 V, respectively. The dCz- and dTC-containing copolymer electrodes displayed different multichromic behaviors with PdCz and PdTC electrodes. P(dCz2-*co*-dTC1) film was yellowish green, gray, and purplish grey at 0.0, 0.6, and 1.1 V, respectively, whereas P(dCz2-*co*-dTC2) film was yellowish green, greenish gray, gray, and purplish gray at 0.0, 0.6, 0.8, and 1.1 V, respectively, and P(dCz1-*co*-dTC2) film was yellow, gray, and dark purplish-grey at 0.0, 0.4, and 1.1 V, respectively. The colorimetric values (L*, a*, and b*), CIE (Commission Internationale de I’Eclairage) chromaticity values (*x*, *y*) and diagrams of the five polymer films at various potentials are listed in Table 2.

### 3.4. Electrochromic Switching of Polymer Electrodes

**Figure 6:**
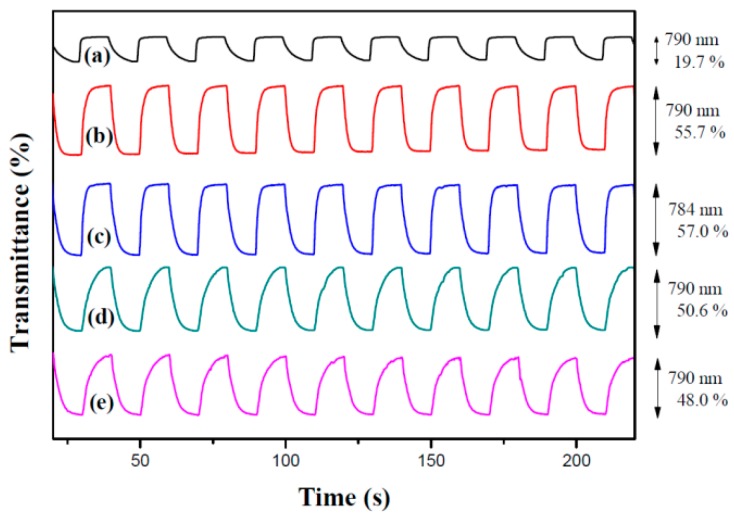
Optical contrast of (**a**) PdCz; (**b**) P(dCz2-*co*-dTC1); (**c**) P(dCz2-*co*-dTC2); (**d**) P(dCz1-*co*-dTC2) and (**e**) PdTC electrodes in an ACN/DCM (1:1, by volume) solution containing 0.2 M LiClO_4_ with a residence time of 10 s.

As displayed in Figure 6, PdCz, P(dCz2-*co*-dTC1), P(dCz2-*co*-dTC2), P(dCz1-*co*-dTC2), and PdTC films were monitored by potential stepping between neutral (0.0 V) and oxidized states (+1.1 V) with a residence time of 10 s. The Δ*T* values of PdCz, P(dCz2-*co*-dTC1), P(dCz2-*co*-dTC2), P(dCz1-*co*-dTC2), and PdTC films were 19.7% at 790 nm, 55.7% at 790 nm, 57.0% at 784 nm, 50.6% at 790 nm, and 48.0% at 790 nm, respectively. The Δ*T* of P(dCz2-*co*-dTC1), P(dCz2-*co*-dTC2), and P(dCz1-*co*-dTC2) films were larger than those of PdCz and PdTC films in 0.2 M LiClO_4_/ACN/DCM solution, indicating copolymers contain both dCz and dTC units increased Δ*T* significantly. The *τ*_c_ of electrodes (a), (b), (c), (d), and (e) were 4.0 s, 2.7 s, 3.5 s, 4.2 s, and 4.3 s at 3rd cycles, respectively, and the *τ*_b_ of electrodes (a), (b), (c), (d), and (e) were 0.8 s, 2.7 s, 1.8 s, 5.6 s, and 6.0 s at 3rd cycles, respectively. Different *τ*_c_ and *τ*_b_ in each set of samples may be attributed to the contact area between electrolytes and electrodes.

ΔOD can be determined using the formula [31],

(1) $$\Delta \mathrm{OD}=\log(\frac{T_{\mathrm{ox}}}{T_{\mathrm{red}}})$$

where *T*_ox_ and *T*_red_ are the transmittances in the oxidized and the reduced state, respectively.

The coloration efficiency (*η*) is another important parameter for practical application of polymer films in ECDs and it can be determined using the equation [32]:

(2) $$\eta =\frac{\Delta \mathrm{OD}}{Q_{d}}$$

where ΔOD is the discrepancy of optical density, *Q*_d_ implicates the amount of injected charges per unit electrode area. The *η* values of PdCz, P(dCz2-*co*-dTC1), P(dCz2-*co*-dTC2), P(dCz1-*co*-dTC2), and PdTC films are 81.4 cm^2^ C^−1^ at 790 nm, 99.3 cm^2^ C^−1^ at 790 nm, 248.4 cm^2^ C^−1^ at 784 nm, 145.3 cm^2^ C^−1^ at 790 nm, and 164.9 cm^2^ C^−1^ at 790 nm, respectively (Table 3). P(dCz2-*co*-dTC2) film presents the highest *η* among the five polymer films.

### 3.5. Spectroelectrochemical Investigation of ECDs

**Figure 7:**
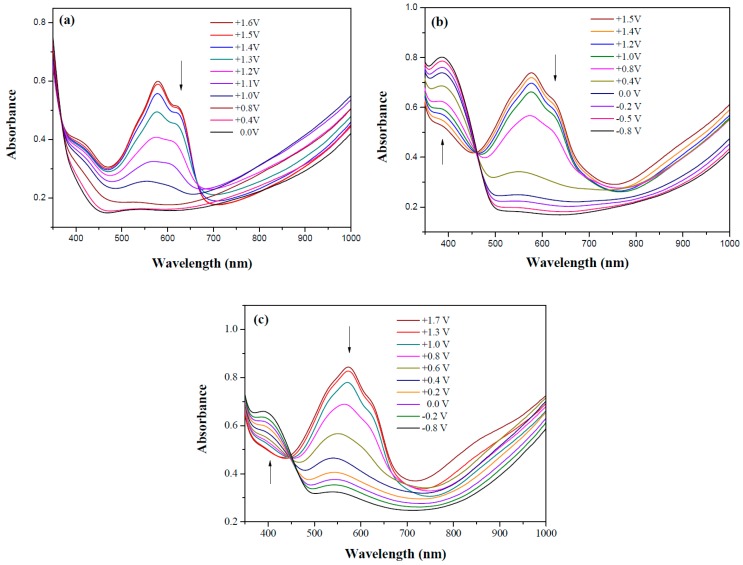
UV-Visible spectra of (**a**) PdCz/PProdot-Me_2_; (**b**) P(dCz2-*co*-dTC2)/PProdot-Me_2_ and (**c**) PdTC/PProdot-Me_2_ ECDs.

Figure 7 displays the UV-Vis spectra of dual-type PdCz/PProdot-Me_2_, P(dCz2-*co*-dTC2)/PProdot-Me_2_, and PdTC/PProdot-Me_2_ ECDs at different applied potentials. 

PdCz/PProdot-Me_2_ ECD did not show distinct absorption peaks at wavelength ranging from 350 to 450 nm at 0.0 V, P(dCz2-*co*-dTC2)/PProdot-Me_2_ and PdTC/PProdot-Me_2_ ECDs displayed distinct absorption peaks at 410 and 402 nm at 0.0 V, which could be assigned as the absorption peaks of P(dCz2-*co*-dTC2) and PdTC films at 0.0 V, respectively. The absorption peaks of P(dCz2-*co*-dTC2) and PdTC films fade with the increasing applied potential, and new absorption bands were emerged gradually at around 580 nm. This can be attributed to the stepwise reduction of PProdot-Me_2_ film. The colors of PdCz/PProdot-Me_2_ ECD are silver gray, gray, and purplish-gray at 0.0, 1.0, and 1.3 V, respectively. P(dCz2-*co*-dTC2)/PProdot-Me_2_ and PdTC/PProdot-Me_2_ ECDs reveal four kinds of colors from bleached to colored states. The colors of P(dCz2-*co*-dTC2)/PProdot-Me_2_ ECD are yellowish green, iron gray, dark blue, and purplish-blue at −0.8, 0.8, 1.2, and 1.5 V, respectively. PdTC/PProdot-Me_2_ ECD reveals yellowish-green, greenish-gray, grayish-blue, and purplish-blue at −0.8, 0.0, 0.8, and 1.7 V, respectively. The electrochromic photographs, colorimetric values (L*, a*, and b*), CIE chromaticity values (*x*, *y*) and diagrams of PdCz/PProdot-Me_2_, P(dCz2-*co*-dTC2)/PProdot-Me_2_, and PdTC/PProdot-Me_2_ ECDs at various applied potentials are listed in Table 4. 

### 3.6. Electrochromic Switching of ECDs

**Figure 8:**
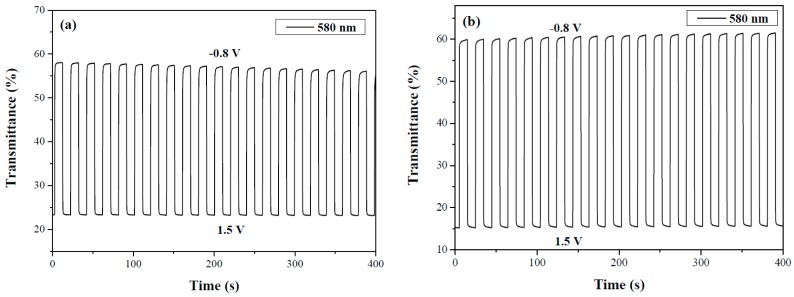
Optical contrast of (**a**) PdCz/PProdot-Me_2_ and (**b**) P(dCz2-*co*-dTC2)/PProdot-Me_2_ ECDs with a residence time of 10 s.

As displayed in Figure 8, PdCz/PProdot-Me_2_ and P(dCz2-*co*-dTC2)/PProdot-Me_2_ ECDs were monitored by potential stepping between bleached and colored states with a residence time of 10 s. The Δ*T*, ΔOD, switching time, and *η* of PdCz/PProdot-Me_2_, P(dCz2-*co*-dTC1)/PProdot-Me_2_, P(dCz2-*co*-dTC2)/PProdot-Me_2_, P(dCz1-*co*-dTC2)/PProdot-Me_2_ and PdTC/PProdot-Me_2_ ECDs are listed in Table 5. P(dCz2-*co*-dTC2)/PProdot-Me_2_ ECD displays the highest Δ*T*, and P(dCz2-*co*-dTC1)/PProdot-Me_2_ and P(dCz2-*co*-dTC2)/PProdot-Me_2_ ECDs display higher Δ*T* and ΔOD than those of PdCz/PProdot-Me_2_ and PdTC/PProdot-Me_2_ ECDs, indicating that the use of copolymers (P(dCz2-*co*-dTC1) and P(dCz2-*co*-dTC2)) as the electrode materials brings about a higher Δ*T* at around 580 nm than those of homopolymers (PdCz and PdTC). Table 6 summarizes comparisons of Δ*T* with reported ECDs, P(dCz2-*co*-dTC2)/PProdot-Me_2_ ECD shows higher Δ*T* than those reported for P(Bmco)/PEDOT [33], P(dNcbph)/PEDOT [34], P(tnCz1-bTp2)/PProdot-Me_2_ [35], p(dNcbph-*co*-bth)/PEDOT [36], PtCz/PProDOT-Me_2_ [37], and P(BCz-*co*-ProDOT)/triple-layer PEDOT-PSS ECDs [38]. The *τ*_c_ and *τ*_b_ of five ECDs in Table 5 are less than 1 s, which are shorter than those of polymer electrodes in a solution, disclosing the ECDs change color faster than the polymers in a solution during stepwise oxidation and reduction period [39]. 

The *η* of PdCz/PProdot-Me_2_, P(dCz2-*co*-dTC1)/PProdot-Me_2_, P(dCz2-*co*-dTC2)/PProdot-Me_2_, P(dCz1-*co*-dTC2)/PProdot-Me_2_ and PdTC/PProdot-Me_2_ ECDs were 507.0, 562.4, 528.8, 438.9 and 440.4 cm^2^ C^−1^, respectively. P(dCz2-*co*-dTC1) film with a dCz/dTC = 2/1 feed molar ratio presented the highest *η* among the five ECDs. Table 6 also summarizes comparisons of *η* with reported ECDs: P(dCz2-*co*-dTC2)/PProdot-Me_2_ ECD showed higher *η* than those reported for p(dNcbph-*co*-bth)/PEDOT [36], PtCz/PProDOT-Me_2_ [37], and P(BCz-*co*-ProDOT)/triple-layer PEDOT-PSS ECDs [38]. However, P(dCz2-*co*-dTC2)/PProdot-Me_2_ ECD showed lower *η* than that reported for P(tnCz1-bTp2)/PProdot-Me_2_ ECD [35]. 

### 3.7. Open-Circuit Memory of ECDs

**Figure 9:**
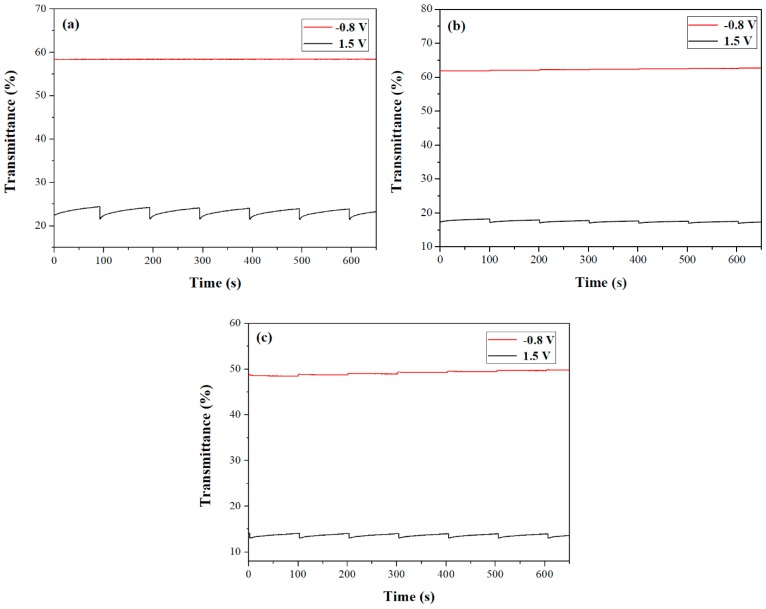
Open circuit stability of (**a**) PdCz/PProdot-Me_2_; (**b**) P(dCz2-*co*-dTC2)/PProdot-Me_2_ and (**c**) PdTC/PProdot-Me_2_ ECDs.

The open-circuit memory effect of PdCz/PProdot-Me_2_, P(dCz2-*co*-dTC2)/PProdot-Me_2_, and PdTC/PProdot-Me_2_ ECDs was detected at bleached and colored states by applying the voltage for 1 s for each 100 s interval [40,41]. Figure 9 shows that the transmittance of the three ECDs was almost no change at the bleached state. At colored states, the three ECDs were less stable than those at bleached state. However, the loss in transmittance of ECDs at colored states was less than 4%. It is worth mentioning that P(dCz2-*co*-dTC2)/PProdot-Me_2_ ECD showed less transmittance change at the colored state than those of PdCz/PProdot-Me_2_ and PdTC/PProdot-Me_2_ ECDs, disclosing that P(dCz2-*co*-dTC2)/PProdot-Me_2_ ECD exhibited a satisfactory open-circuit memory effect.

### 3.8. Electrochemical Stability of ECDs

**Figure 10:**
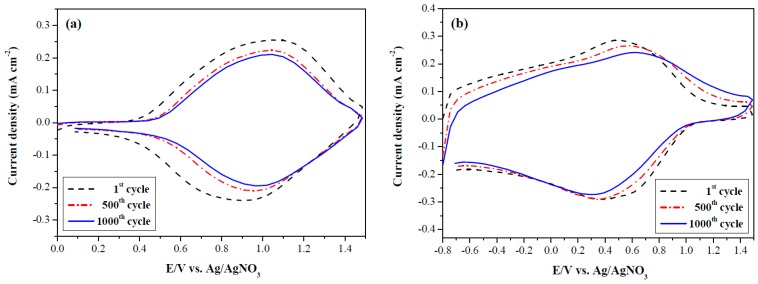

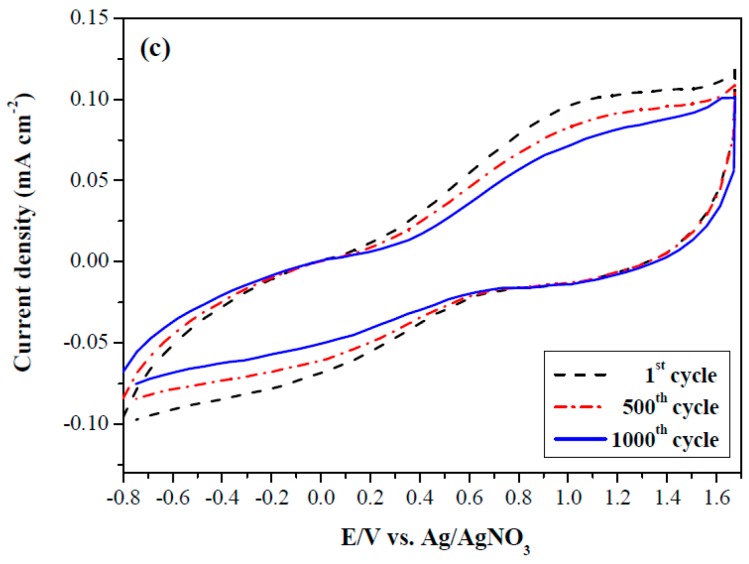
Cyclic voltammograms of (**a**) PdCz/PProdot-Me_2_; (**b**) P(dCz2-*co*-dTC2)/PProdot-Me_2_ and (**c**) PdTC/PProdot-Me_2_ ECDs at a scan rate of 500 mV s^−1^ between one and 1000 cycles.

The long-term electrochemical stability of PdCz/PProdot-Me_2_, P(dCz2-*co*-dTC2)/PProdot-Me_2_, and PdTC/PProdot-Me_2_ ECDs was monitored using CV at the 1st, 500th and 1000th cycles [42]. From the observation of ECDs’ electrochemical ability in Figure 10, 78.4%, 95.5%, and 87.7% of electroactive stability were preserved at the 500th cycle, and 72.5%, 88.0%, and 78.2% of electroactive stability were preserved at the 1000th cycle for PdCz/PProdot-Me_2_, P(dCz2-*co*-dTC2)/PProdot-Me_2_, and PdTC/PProdot-Me_2_ ECDs, respectively. P(dCz2-*co*-dTC2)/PProdot-Me_2_ ECD showed better long-term electrochemical stability than those of PdCz/PProdot-Me_2_ and PdTC/PProdot-Me_2_ ECDs, disclosing the redox stability of copolymerized P(dCz2-*co*-dTC2) film was higher than those of homopolymerized PdCz and PdTC films and ECD employed a copolymer (P(dCz2-*co*-dTC2)) as anodic polymer film gave rise to a better long-term electrochemical stability than those of homopolymers (PdCz and PdTC).

## 4. Conclusions

Five ECDs’ polymer electrodes (PdCz, P(dCz2-*co*-dTC1), P(dCz2-*co*-dTC2), P(dCz1-*co*-dTC2), and PdTC) were prepared using electrochemical copolymerization. Our experimental studies display that dCz- and dTC-containing copolymer electrodes showed multichromic behaviors and the electrochromic switching properties and coloration efficiency of the copolymers could be adjusted by various feed monomer molar ratios. P(dCz2-*co*-dTC1) film was yellowish green, gray, and purplish grey at 0.0, 0.6, and 1.1 V, respectively, whereas P(dCz2-*co*-dTC2) film was yellowish green, greenish gray, gray, and purplish gray at 0.0, 0.6, 0.8, and 1.1 V, respectively. P(dCz2-*co*-dTC2) film displayed a high Δ*T* (57.0 %) and a high *η* (248.4 cm^2^ C^−1^) at 784 nm in 0.2 M LiClO_4_/ACN/DCM solution. Five ECDs based on dCz- and dTC-containing anodic polymer electrodes and PProdot-Me_2_ cathodic polymer electrodes were built and electrochromic properties were characterized. P(dCz2-*co*-dTC2)/PProdot-Me_2_ ECD exhibited a high Δ*T* (45.8 % at 580 nm) and high long-term electrochemical stability, whereas P(dCz2-*co*-dTC1)/PProdot-Me_2_ ECD exhibited high *η* (562.4 cm^2^ C^−1^ at 580 nm) and a rapid switching time (≤0.3 s). In view of the above studies, dCz- and dTC-containing copolymers are amenable for use in ECDs.

## Figures and Tables

**Table 1:** Feed species and molar ratio of anodic polymer electrodes (a)–(e).

Electrodes	Anodic Polymer	Feed Species of Anodic Polymer	Feed Molar Ratio of Anodic Polymer
(a)	PdCz	2 mM dCz	Neat dCz
(b)	P(dCz2-*co*-dTC1)	2 mM dCz + 1 mM dTC	2:1
(c)	P(dCz2-*co*-dTC2)	2 mM dCz + 2 mM dTC	2:2
(d)	P(dCz1-*co*-dTC2)	1 mM dCz + 2 mM dTC	1:2
(e)	PdTC	2 mM dTC	Neat dTC

**Table 2:** Colorimetric values (L*, a*, and b*), CIE chromaticity values (*x*, *y*) and diagrams of the (**a**) PdCz, (**b**) P(dCz2-*co*-dTC1), (**c**) P(dCz2-*co*-dTC2), (**d**) P(dCz1-*co*-dTC2), and (**e**) PdTC at various applied potentials.

(a)						
**Potential (V)**	L*	a*	b*	***x***	***y***	**Diagram**
0.0	95.12	−0.34	2.95	0.4500	0.4106	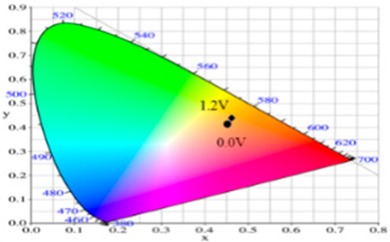
0.8	92.64	−1.33	18.74	0.4638	0.4258	
0.9	90.35	−1.99	25.66	0.4692	0.4328	
1.0	87.76	−3.07	23.96	0.4664	0.4334	
1.2	84.27	−4.52	18.11	0.4588	0.4311	
(b)						
**Potential (V)**	L*	a*	b*	***x***	***y***	**Diagram**
−0.5	90.36	−2.48	41.83	0.4815	0.4456	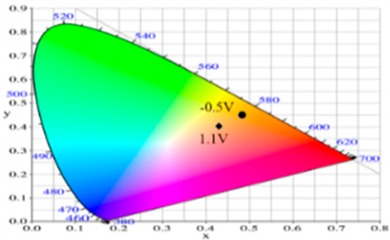
0.2	78.82	1.71	24.79	0.4775	0.4293	
0.4	64.49	5.65	1.11	0.4612	0.3999	
0.8	56.45	6.50	−7.41	0.4509	0.3859	
1.1	58.22	−2.34	−8.72	0.4284	0.3987	
(c)						
**Potential (V)**	L*	a*	b*	***x***	***y***	**Diagram**
−0.2	87.82	−1.08	35.11	0.4793	0.4395	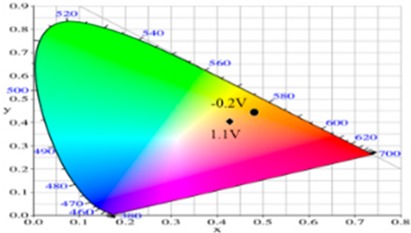
0.2	65.75	4.92	1.02	0.4593	0.4011	
0.8	57.30	5.37	−6.45	0.4498	0.3893	
0.9	56.84	3.41	−6.98	0.4444	0.3916	
1.1	57.99	−3.38	−8.82	0.4258	0.4002	
(d)						
**Potential (V)**	L*	a*	b*	***x***	***y***	**Diagram**
−0.2	85.42	0.44	42.42	0.4883	0.4432	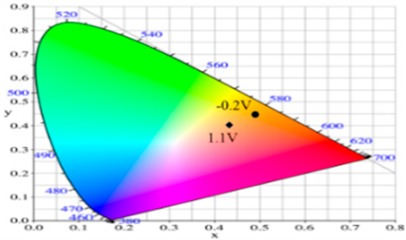
0.2	75.20	3.48	26.48	0.4836	0.4287	
0.6	54.48	7.37	−6.51	0.4546	0.3851	
0.9	53.03	4.21	−6.75	0.4466	0.3898	
1.1	55.04	−1.72	−7.61	0.4309	0.3990	
(e)						
**Potential (V)**	L*	a*	b*	***x***	***y***	**Diagram**
−0.2	87.95	−1.58	31.51	0.4754	0.4374	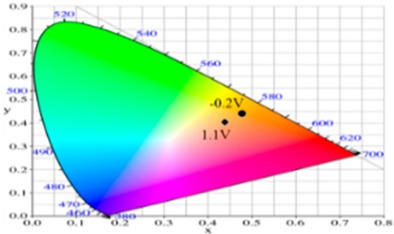
0.2	82.07	0.27	22.50	0.4719	0.4287	
0.6	65.32	5.12	−2.87	0.4543	0.3960	
0.9	61.22	5.69	−5.11	0.4525	0.3915	
1.1	61.51	−0.69	−6.32	0.4366	0.3999	

**Table 3:** Optical and electrochemical properties investigated at the selected applied wavelength for the electrodes.

Electrodes	λ (nm)	*T*_ox_	*T*_red_	Δ*T*	ΔOD	Q*_d_* (mC cm^−^^2^)	*η* (cm^2^ C^−^^1^)	τ_c_ (s)	τ_b_ (s)
PdCz	790	69.7	89.3	19.7	0.11	1.351	81.4	4.0	0.8
P(dCz2-*co*-dTC1)	790	18.5	73.9	55.7	0.60	6.043	99.3	2.7	2.7
P(dCz2-*co*-dTC2)	784	10.7	67.7	57.0	0.80	3.221	248.4	3.5	1.8
P(dCz1-*co*-dTC2)	790	10.9	61.4	50.6	0.75	5.163	145.3	4.2	5.6
PdTC	790	9.1	57.1	48.0	0.80	4.851	164.9	4.3	6.0

**Table 4:** Electrochromic photographs, colorimetric values (L*, a*, and b*), CIE chromaticity values (*x*, *y*) and diagrams of the PdCz/PProdot-Me_2_, P(dCz2-*co*-dTC2)/PProdot-Me_2_ and PdTC/PProdot-Me_2_ ECDs at various applied potentials.

ECDs	Potential (V)	Photographs	L*	a*	b*	*x*	*y*	Diagrams
PdCz/PProdot-Me_2_	0.0	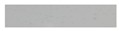	86.69	0.03	−0.06	0.4476	0.4073	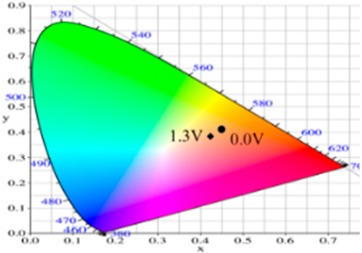
	0.8	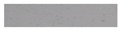	84.93	−0.01	3.78	0.4517	0.4113	
	1.0	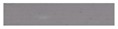	75.69	1.54	−3.55	0.4460	0.4013	
	1.2	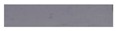	67.20	0.73	−14.09	0.4290	0.3881	
	1.3	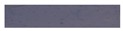	63.53	1.25	−18.88	0.4216	0.3796	
P(dCz2-*co*-dTC2)/PProdot-Me_2_	−0.8	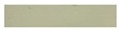	85.06	−1.40	26.92	0.4724	0.4342	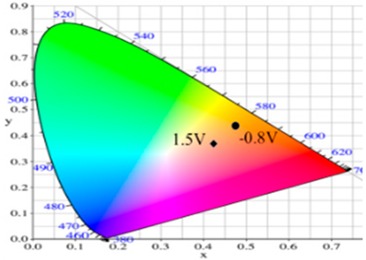
	0.0	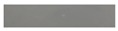	80.27	−0.28	19.04	0.4679	0.4268	
	0.8	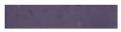	61.82	2.72	−8.38	0.4410	0.3918	
	1.2	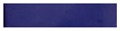	56.30	4.46	−17.66	0.4284	0.3737	
	1.5	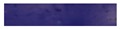	54.18	5.26	−21.30	0.4229	0.3655	
PdTC/PProdot-Me_2_	−0.8	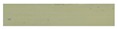	75.67	2.71	13.63	0.4686	0.4179	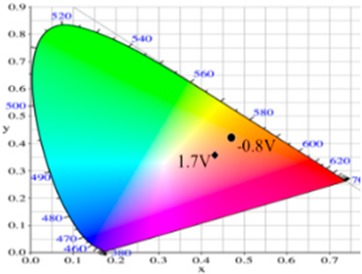
	0.0	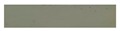	72.35	3.54	8.02	0.4644	0.4112	
	0.8	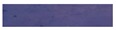	55.56	8.38	−14.34	0.4433	0.3724	
	1.3	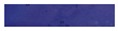	50.05	9.67	−22.05	0.4307	0.3547	
	1.7	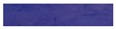	49.19	9.66	−22.34	0.4298	0.3535	

**Table 5:** Optical and electrochemical properties investigated at the selected applied wavelength for the devices.

Devices	λ (nm)	*T*_ox_ (V ^a^)	*T*_red_ (V ^a^)	Δ*T*	ΔOD	Q*_d_* (mC cm^−2^)	*η* (cm^2^ C^−1^)	*τ*_c_ (s)	*τ*_b_ (s)
PdCz/PProdot-Me_2_	580	23.1 (1.5)	57.5 (−0.8)	34.4	0.396	0.781	507.0	0.2	0.2
P(dCz2-*co*-dTC1)/PProdot-Me_2_	580	15.1 (1.5)	55.2 (−0.8)	40.1	0.563	1.001	562.4	0.3	0.2
P(dCz2-*co*-dTC2)/PProdot-Me_2_	580	16.0 (1.5)	61.8 (−0.8)	45.8	0.587	1.110	528.8	0.2	0.3
P(dCz1-*co*-dTC2)/PProdot-Me_2_	578	36.8 (1.5)	69.0 (−0.8)	32.2	0.273	0.622	438.9	0.9	0.2
PdTC/PProdot-Me_2_	578	15.6 (1.5)	46.8 (−0.8)	31.2	0.477	1.083	440.4	0.2	0.2

^a^ The selected applied voltages for the devices.

**Table 6:** Optical contrast and coloration efficiencies of some ECDs.

ECD Configuration	Δ*T*_max_ (%)	*η* (cm^2^ C^−1^)	Ref.
P(Bmco)/PEDOT	35 (620 nm)	---	[33]
P(dNcbph)/PEDOT	19 (550 nm)	---	[34]
P(tnCz1-bTp2)/PProdot-Me_2_	40 (630 nm)	539 (630 nm)	[35]
p(dNcbph-*co*-bth)/PEDOT	28.6 (700 nm)	234 (700 nm)	[36]
PtCz/PProDOT-Me_2_	36 (572 nm)	343.4 (572 nm)	[37]
P(BCz-*co*-ProDOT)/triple-layer PEDOT-PSS	41 (642 nm)	417 (642 nm)	[38]
P(dCz2-*co*-dTC2)/PProdot-Me_2_	45.8 (580 nm)	528.8 (580 nm)	This work
